# Employing Atomic Force Microscopy (AFM) for Microscale Investigation of Interfaces and Interactions in Membrane Fouling Processes: New Perspectives and Prospects

**DOI:** 10.3390/membranes14020035

**Published:** 2024-01-27

**Authors:** Mohan Wei, Yaozhong Zhang, Yifan Wang, Xiaoping Liu, Xiaoliang Li, Xing Zheng

**Affiliations:** 1 State Key Laboratory of Eco-hydraulics in North West Arid Region, Xi’an University of Technology, Xi’an 710048, China; weimh1998@163.com (M.W.); yfanw23@163.com (Y.W.); liuxiaoping0111@163.com (X.L.); lixiaoliang@xaut.edu.cn (X.L.); 2 Yulin Coal Chemical Waste Resource Utilization and Low Carbon Environmental Protection Engineering Technology Research Center, Yulin High-tech Zone Yuheng No. 1 Industrial Sewage Treatment Co., Ltd., Yulin 719000, China

**Keywords:** Atomic force microscopy (AFM), membrane fouling, morphology, roughness, interactions

## Abstract

Membrane fouling presents a significant challenge in the treatment of wastewater. Several detection methods have been used to interpret membrane fouling processes. Compared with other analysis and detection methods, atomic force microscopy (AFM) is widely used because of its advantages in liquid-phase in situ 3D imaging, ability to measure interactive forces, and mild testing conditions. Although AFM has been widely used in the study of membrane fouling, the current literature has not fully explored its potential. This review aims to uncover and provide a new perspective on the application of AFM technology in future studies on membrane fouling. Initially, a rigorous review was conducted on the morphology, roughness, and interaction forces of AFM in situ characterization of membranes and foulants. Then, the application of AFM in the process of changing membrane fouling factors was reviewed based on its in situ measurement capability, and it was found that changes in ionic conditions, pH, voltage, and even time can cause changes in membrane fouling morphology and forces. Existing membrane fouling models are then discussed, and the role of AFM in predicting and testing these models is presented. Finally, the potential of the improved AFM techniques to be applied in the field of membrane fouling has been underestimated. In this paper, we have fully elucidated the potentials of the improved AFM techniques to be applied in the process of membrane fouling, and we have presented the current challenges and the directions for the future development in an attempt to provide new insights into this field.

## 1. Introduction

Membrane separation technology has emerged as the preferred method for producing clean water during wastewater treatment and desalination. This preference is attributed to the high separation accuracy, energy efficiency, lack of secondary pollution, and ease of operation of the technology [1,2]. Membrane fouling is a key obstacle in membrane applications, including ultrafiltration (UF), microfiltration (MF), nanofiltration (NF), and reverse osmosis (RO) [3]. Membrane fouling is a particularly serious problem in the pre-treatment processes of industrial wastewater, leading to poor water quality and increased operating costs [4,5]. A thorough understanding of fouling formation and properties is required in wastewater treatment using membranes and contributes to slowing down membrane fouling and implementing appropriate control measures. In response, extensive foundational investigations of membrane fouling have been conducted, with researchers seeking to clarify primary foulants, membrane–foulant interactions, and potential fouling mitigation techniques.

**Figure 1:**
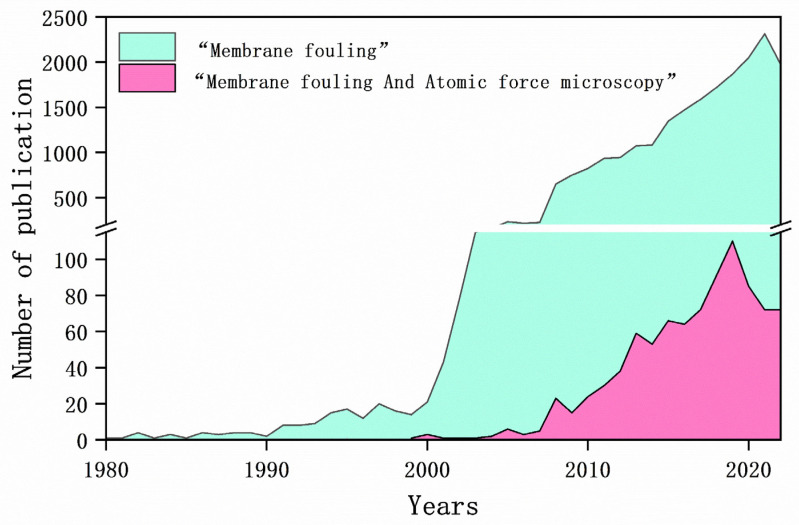
Membrane fouling and atomic force microscopy research published since 1980. The graph depicts the number of publications related to “membrane fouling” (blue) or “membrane fouling and atomic force microscopy” (pink) from 1980 to 2022.

Figure 1 shows the annual number of publications related to membrane fouling since 1980 (represented by the blue segment). In 2021, a total of 2313 publications on membrane fouling underscored the keen interest in the discipline. The formation of membrane fouling is complex and microscopic, and the factors involved are variable and complex. The membrane properties and foulants, mechanical properties, and solution properties could all affect the microscopic interaction of fouling. Comprehensive microscopic analysis of membrane fouling relies on sophisticated analytical techniques.

Atomic force microscopy (AFM), first introduced in 1986, has emerged as a vital analytical technique for investigating the surface structure of solid materials, including insulators. This technique is used widely to study membrane fouling. The increase in publications related to AFM application in membrane fouling since 2000, as shown in Figure 1 (pink portion), underscores the increasing interest of researchers. Advancement in AFM technology has led to it becoming an indispensable tool for scrutinizing membrane surface morphology and fouling characteristics [6].

Currently, both AFM and scanning electron microscopes are used extensively for surface analyses. However, compared with SEM, AFM offers superior imaging resolution and achieving nanoscale 3D imaging. Sample preparation for SEM requires complex processes, such as metal coating, which can distort gel-like films, whereas AFM typically involves in situ measurements and it is compatible with liquid environments, only requiring the sample to be positioned on a substrate for imaging. Furthermore, AFM operates at room temperature and pressure and is suitable for both conductive and nonconductive materials, whereas SEM requires a vacuum environment and it is only appropriate for conductive samples. In addition to imaging, AFM can detect mechanical properties. In summary, AFM is an exceptionally powerful technique for analyzing membrane surfaces in liquid environments or situations requiring higher vertical resolution.

**Figure 2:**
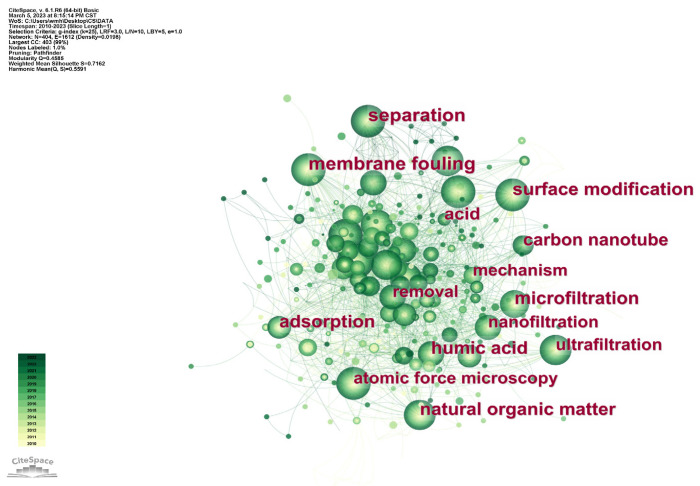
Analysis of keywords related to membrane fouling and atomic force microscopy research.

A visual analysis of the articles related to “membrane fouling and atomic force microscopy” is shown in Figure 2, which were extracted from “Web of Science” database and analyzed by CiteSpace software(6.2.R6). The analysis reveals high-frequency keywords, such as “surface modification”, “humic substances”, “nanofiltration”, “microfiltration”, and “natural organic matter”, signifying the extensive application of AFM in diverse aspects of membrane fouling research. Typically, AFM is employed to characterize the surface morphology and roughness of both membranes and foulants [7,8,9], as well as to quantify surface adhesion forces using AFM colloidal probes [10,11], helping to understand membrane fouling from a microscopic perspective and explore the mechanism of membrane fouling. This makes a significant contribution to the research on membrane fouling.

Prior to this, little of the literature explored the possibility of AFM applications in the field of membrane fouling. Utilizing AFM for microscale investigations of the interfaces and interactions in membrane fouling processes helps reveal the mechanisms of fouling and optimize control strategies. Building upon this foundation, the present study offers a comprehensive review of the literature on AFM characterization of membranes and foulants, delving deeper into the viability of employing AFM for interfacial research on membrane fouling. The remainder of this paper is organized as follows. The first section provides the background and motivation for the review and briefly describes the application of AFM in membrane fouling. The second section provides a concise introduction to AFM technology. The third part delves deeper into the application of AFM in membrane fouling, focusing on its use in characterizing the morphology, roughness, channels of membranes and foulants, and highlights the advantages of AFM in measuring the interaction force between membranes and foulants. Specifically, our research team employed AFM to conduct a series of in situ measurements, continuously observing the dynamic changes in membrane contamination under varying conditions such as salt concentration, pH, duration, and electric field and voltage (Section 3.3). We elaborate on the efforts and studies conducted by our group in this area and share our insights and in-depth reflections. In this section, where the theory and models of interactions during membrane fouling are discussed. AFM measurements of membrane fouling or foulant–foulant interaction forces can validate the fitting results of the interaction force calculation model.

In addition, with the development of AFM technology, a number of improved AFM techniques have emerged, and the potential of these improved AFM techniques to be applied in the field of membrane fouling has been underutilized in the field of membrane fouling. In this paper, we have fully elucidated the potential of the improved AFM techniques to be applied in the process of membrane fouling. The fourth part discusses cutting-edge AFM technologies currently under development, such as AFM with modified probes, combined with different functional modules, coupled with other technologies, and the application of video-grade HS-AFM in membrane fouling, providing insights into future perspectives for the development of AFM in this field. This review elaborates on the current state of AFM research regarding membrane fouling with the objective of providing readers with an enriched understanding of the status, progress, and future development trajectories of AFM technology within the membrane fouling domain.

## 2. Introduction to AFM

**Figure 3:**
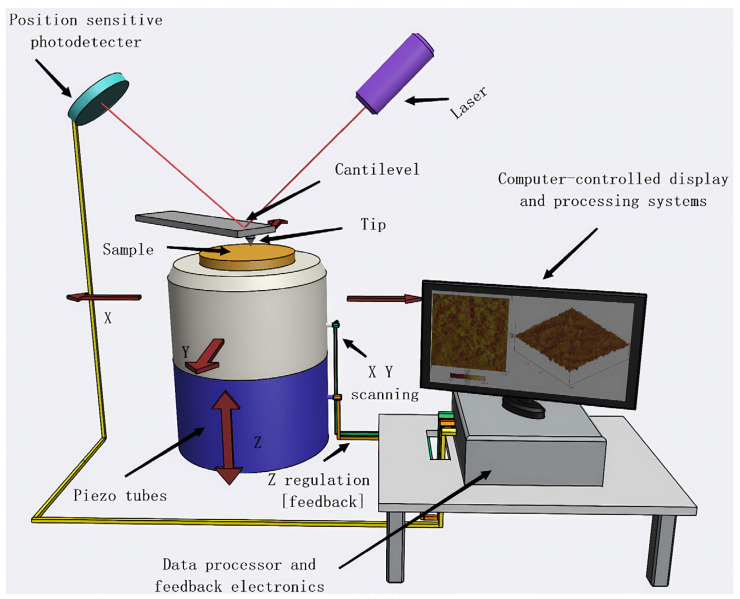
Schematic diagram of an atomic force microscope (AFM).

As shown in Figure 3, an AFM mainly comprises a microcantilever with a sharp tip, microcantilever motion detection system, feedback loop, piezoelectric ceramic scanning mechanism, and computer-controlled system for image acquisition, display, and processing. When the tip approaches the sample, short-range repulsive forces emerge between them, enabling the capture of atomic-scale resolution images of the surface.

The AFM technique is commonly used to obtain three-dimensional (3D) images of sample surfaces and discern nanoscale features, surpassing other high-resolution imaging techniques [12]. A significant advantage of AFM is its ability to examine samples in a non-vacuum, real liquid, or air environments [13], which is crucial for exploring sample surfaces and contamination under specific environmental conditions. Moreover, AFM provides various imaging modes [14], each with unique benefits. Contact mode, which is relatively straightforward to operate, is generally used to image hard, planar surfaces. The tapping or noncontact mode [15] involves continuous oscillation of the cantilever near the sample surface, allowing the sharp tip to intermittently make contact with the surface. This approach overcomes the issues associated with direct contact mode imaging. It is frequently used for imaging soft, easily deformable, or delicate samples and is particularly well-suited for measuring membrane surfaces and membrane fouling under normal conditions.

The AFM technique can be used to characterize morphology, roughness, and membrane channels [7,8,9]. Quantitative analysis of phase images can be conducted to elucidate macromolecular accumulation on the membrane surface and correlate it with membrane performance. Moreover, AFM can quantify the surface adhesion force of the sample using colloidal probes [10,11]. This technique is not only applicable for characterizing membrane and contaminant interfaces, but also for observing contaminated membrane surfaces and quantifying the interaction forces between the contaminants and the membrane interfaces. Utilizing AFM to characterize membrane interfaces and their contamination and analyzing the outcomes aids in comprehensive understanding of membrane contamination interfaces and their interactions.

## 3. Application of AFM in Membrane Fouling

In practical applications, the AFM technology can be used to investigate the fouling processes of UF, NF, RO, and ED membranes. The technology enables the characterization of membrane and foulant interfaces, measurement of surface topography and roughness, and extraction of information about membrane pore sizes, all these factors are vital for understanding fouling mechanisms in wastewater treatment and desalination processes. During the membrane treatment processes, a confluence of factors, including adhesion, hydrogen bonding, van der Waals forces, hydrophobicity, and chemical bonding, contribute to membrane fouling. The interaction between the membrane surface and the foulants determines the ease of foulant removal. The characteristics of both the membrane and the foulant interfaces could influence membrane fouling. Interfacial environmental factors such as aqueous solutions and atmospheric conditions significantly affect the fouling process. Measurements are conducted in isolated environments in several instances. For instance, scanning tunneling, scanning electron, and transmission electron microscopy typically require vacuum conditions for measurements that could cause the sample losing its original state. In contrast, AFM can measure under various environments such as aqueous solutions and atmospheric conditions to provide comprehensive information. The mechanical measurements of AFM contribute to obtaining a deeper understanding of interactions generated at the membrane surface, which is essential for revealing membrane fouling mechanisms.

### 3.1. Characterization of Membrane

**Figure 4:**
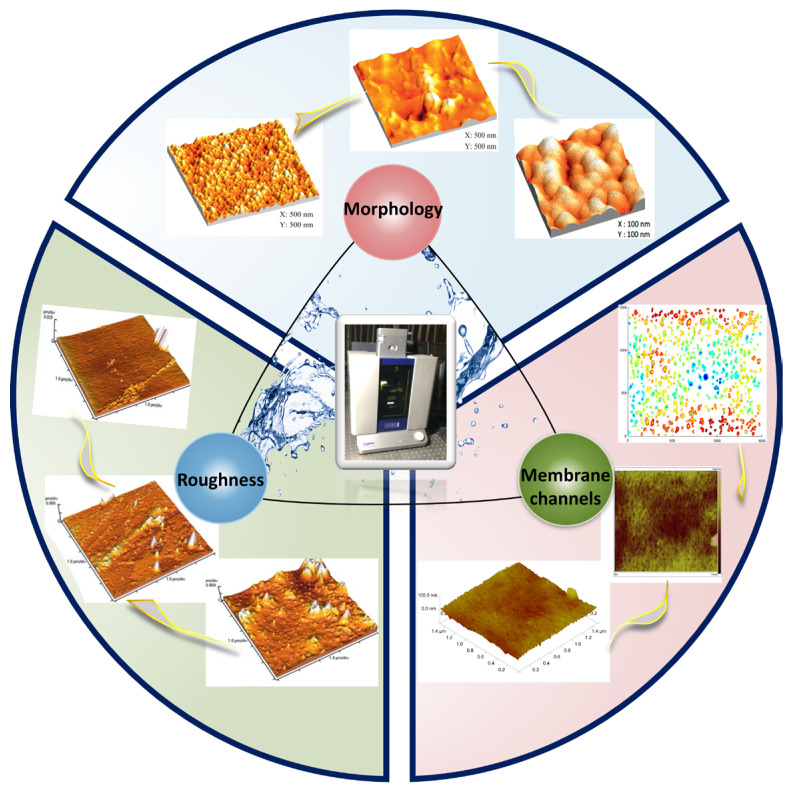
The AFM characterizes several aspects of the membrane, where the AFM images are reproduced from [16,17,18], with permission from Elsevier academic publishing company.

The interaction between the membranes and foulants is related to the membrane surface properties, such as hydrophilicity, roughness, and charge. The development of high-quality antifouling membranes could reduce the interaction forces between foulants and membrane surfaces and decelerate membrane fouling. The technique facilitates the visualization of membrane surfaces at high resolution, characterizing three-dimensional presentation of membrane surface information, allowing for an exhaustive detailed expression of the surface characteristics of the membrane. Figure 4 illustrates several aspects of AFM for membrane characterization.

#### 3.1.1. Characterization of Membrane Morphology

Visualization of the membrane surface morphology aids in understanding the relevant properties of the membrane. AFM excels in visualizing morphological features, allowing for the in situ characterization of morphological changes occurring at the interface of functional layers on membrane surfaces during the polymerization process. During the phase inversion process, utilizing AFM to scan nanofiltration or reverse osmosis membranes prepared under various parameters in a liquid environment enables the in situ observation of more detailed and systematic changes in the surface functional layers [16]. Further, AFM can be used to investigate the degradation effects of soil microbial communities on polyethylene membranes by observing changes in the microstructure of the membrane [19]. Using AFM, it is also possible to observe the ion transport channels of membranes. Examination of modified anion exchange membrane (AEM) materials with densely grafted ionic clusters through atomic force microscopy can reveal distinct ion conduction pathways and demonstrate that the modified AEMs exhibit excellent nano-phase separation [20]. By studying the surfaces of nanofiltration membranes using AFM in different imaging modes, various AFM imaging mode characteristics can be obtained [14]. The tapping mode in AFM allows for precise measurement of the 3D structure of soft and delicate surfaces without damaging their morphology. This technique is particularly useful for studying the interfacial polymerization process of active functional layers on nanofiltration membrane surfaces [6].

Evidently, AFM can be used to understand membrane layer surface smoothness and uniformity; capture membrane surface microscopic morphology, including surface defects, nanoscale protrusions, or depressions; and provide intuitive images of membrane surface morphology. Thus, AFM is not only capable of in situ measurement of surface morphology changes induced by hydrochemical conditions, but also enables the understanding and discovery of ion transport channels and nanoscale morphologies through 3D visualization images. Particularly, the tapping mode is almost non-destructive to thin and soft membrane surfaces. Additionally, AFM can be used to study the surface potential signals of membrane materials, overlaying them with the physicochemical properties of specific areas. These aspects are crucial for characterizing membrane performance and understanding membrane fabrication.

#### 3.1.2. Characterization of Roughness

The surface roughness of a membrane is a key factor influencing interfacial performance and fouling processes. Using AFM not only allows for the observation of a membrane’s surface morphology, but due to its three-dimensional measuring capabilities along the x, y, and z axes, AFM can also precisely characterize in situ the roughness of the membrane surface/functional layer and provide detailed 3D surface topography maps. AFM can be used to understand the roughness of various types of membranes, such as cation exchange membranes. During long-term operation, AFM can precisely measure changes in roughness, thereby establishing the relationship between roughness and ion exchange performance. This enhances the monitoring of the ion exchange effectiveness and contamination level of cation exchange membranes [21].

In the process of membrane modification, the incorporation of specific active components, such as surfactants or polymer monomers, in the adsorption crosslinking process can increase surface roughness and alter the structure of the membrane’s functional layer. Similarly, modifications with carbon nanotubes, metal oxides, and other substances can change the membrane’s morphology, increase roughness, and expand membrane channels, thereby altering the membrane’s performance. Using AFM, changes in membrane surfaces and channels can be observed, and the roughness can be accurately measured. Roughness serves as an important parameter in membrane fabrication. The results generally show that the surface of the original membrane is low in roughness, uniform, and smooth. When modifiers or carbon nanotubes are added, nanoscale modified structures are formed, increasing the roughness. This enhancement in roughness can improve the membrane’s anti-fouling properties, permeation evaporation performance, ion selectivity, and regulate the membrane’s hydrophilicity or hydrophobicity. Asymmetric polystyrene membranes manufactured using the wet phase inversion method with the addition of surfactant (Pluronic F127) increase the surface roughness of the membrane and strengthen the membrane channels, significantly enhancing the permeation evaporation performance of the membrane [22]. Similarly, analysis of multi-walled carbon nanotube (MWCNT) dispersed PS nanofiltration membranes [17] using AFM shows that the surface of the original PS membrane is smooth and uniform, while the addition of MWCNTs increases the surface roughness and makes the structure more evident, thus improving the hydrogen permeability of the PS membrane. Roughness measurements using AFM reveal that plasma treatment and surface acidification also increase the roughness of ion exchange membranes, enabling more ion exchange and facilitating the preparation of ion exchange membranes with excellent performance [23].

However, studies show that for rough surfaces, nanoscale modified structures have better tendencies to prevent membrane fouling, but when the modified structures are too large, they can exacerbate membrane fouling. These related insights can be obtained through the precise measurement of roughness using AFM. In addition, modifying the membrane surface through copolymerization and grafting methods can increase membrane roughness, and AFM can measure roughness changes during the membrane modification process in situ. When characterizing hydrophilic polymer-functionalized polysulfone (PSF) blend membranes using AFM [24], researchers found that the addition of 4VP side chains enhanced the surface roughness of the modified membrane. In another study, PSF membranes modified with titanium oxide compounds [25] had higher surface roughness, and these modified membranes exhibited excellent hydrophilicity and anti-fouling properties. Simple coding of the surfactants polydopamine and 3-(N, N-dimethyl myristoyl) propane sulfonate [26] can achieve anti-fouling properties of polyethersulfone (PES) ultrafiltration membranes. Scanning these modified membranes with AFM can obtain roughness parameters Rq and Ra, indicating that the modified membranes significantly mitigate flux decline and enhance anti-fouling performance.

Precise measurement of membrane surface roughness with AFM can be used to explore which roughness is more resistant to contamination on the modified membrane surface, thus achieving membrane performance adjustment. Although many studies have shown that increased roughness may lead to increased tendencies for membrane fouling [21,24], other research findings suggest that adding micrometer to nanometer-sized particles to increase surface roughness (similar to lotus leaf biomimetic structures) can reduce membrane fouling [17,23]. This discrepancy is mainly because a single roughness parameter is insufficient to summarize the complexity of membrane surface fouling. Establishing a relationship between roughness R measured with AFM and the roughness index H can more quickly and accurately evaluate membrane surface roughness [27]. In addition, an overall assessment should combine AFM with various other techniques to carefully examine membrane surface characteristics, such as surface potential, hydrophilicity/hydrophobicity, functional groups, and foulant properties. Through this comprehensive judgment of characterization results, the relationship between surface roughness and membrane fouling can be thoroughly analyzed. Establishing the relationship between membrane surface roughness measured by AFM and membrane surface potential, functional groups, etc., can better help optimize membrane hydrophilicity/hydrophobicity, permeation selectivity, ion selectivity, and anti-fouling properties, providing guidance for the design and optimization of membrane interfaces.

#### 3.1.3. Measurement of Membrane Channels

Membrane channels are crucial for the filtration performance of membranes, as the size and structure of these channels directly influence the membrane’s selectivity and permeation flux, which relates to the trade-off effect of the membrane. AFM has been utilized to detect various surface parameters of modified membranes, including the structure and pore size of membrane channels. These researchers [18,28,29,30,31,32] characterized the surfaces of modified membranes using AFM and found that the deposition process of modifiers made the membrane surfaces smoother, eliminating small-scale rough features, reducing pore sizes, and decreasing sensitivity to foulants. For instance, Kim et al. [28] achieved atomic-level surface functionalization of nanofiltration membranes using graphene oxide (GO) combined with plasma-enhanced atomic layer deposition (ALD) technology. A novel data analysis method [18] integrating AFM with “pore reconstruction technology” was used to assess membrane channel structures, including size, shape, and interlayer distances. The obtained membrane channel information is vital for the permeation selection process in membrane desalination and can also serve as a crucial factor in assessing the propensity for membrane fouling. AFM can precisely measure the interlayer distances of zinc oxide-coated aluminum membrane channels modified by deposition methods [29], identify the membrane channel structures of superhydrophilic copper mesh membranes coated with zinc oxide nanostructures (ZnO NW) used for oil–water separation [30], acquire information about the shape of membrane channels in modified seawater desalination nanofiltration membranes created using molecular layer deposition (MLD) techniques [31], and obtain 3D shape information of membrane channels in chitosan and polystyrene sulfonate-modified polyamide microfiltration membranes prepared by layer-by-layer (LBL) deposition methods [32].

Combining the surface and cross-sectional images obtained from AFM can construct three-dimensional images of membrane channels, providing detailed information on the size, shape, and arrangement of channels, and more accurately predicting the degree of membrane channel clogging and membrane fouling. Studies indicate that uniformly distributed pore structures, as opposed to uneven distributions, are likely to reduce the risk of fouling. The systematic distribution of pores in uniform membranes can enhance the interception capacity for foulants [33]. Notably, the geometric shape of membrane channels greatly influences membrane fouling. Typically, the fouling intensity caused by slit-shaped pores is lower than that caused by circular pores [34]. Additionally, AFM can accurately present the degree of membrane channel clogging and the state of membrane fouling in real liquid-phase environments. In contrast, SEM requires measurements under dry and vacuum conditions, which may lead to distorted results and inaccurate pore information. Therefore, membrane channel data obtained through AFM are crucial for tracking membrane fouling trajectories and assessing and improving membrane performance.

**Figure 5:**
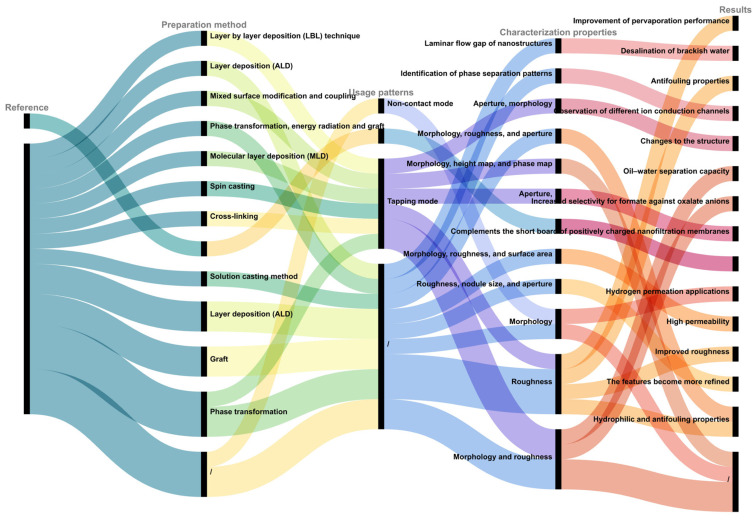
Different aspects and results of AFM characterization of modified membranes in different modes.

As previously mentioned, employing AFM to characterize different membranes allows for a more comprehensive observation of three-dimensional surface morphology, membrane roughness measurements, membrane channel assessments, and an overall evaluation of the material characteristics and application performance of membranes and modified membranes. As depicted in Figure 5, this section categorizes and summarizes the different modes, characteristics, and outcomes of modified membrane characterization via AFM as referenced in the mentioned literature. AFM has also been extensively applied in characterizing modified membranes of various materials, such as modified Langmuir–Blodgett (LB) thin films [35], uniquely shaped modified block copolymer microfiltration membranes [36], zeolite-filled polyethersulfone membranes [37], modified Carbosep M5 ceramic membranes [38], innovative positively charged nanofiltration membranes [39], organic membranes for oil–water separation [40], and composite ceramic microfiltration membranes for greywater treatment [41]. This technique (atomic force microscopy) has become a powerful tool in the design and fabrication of functional membranes.

### 3.2. Characterization of Contaminants

**Figure 6:**
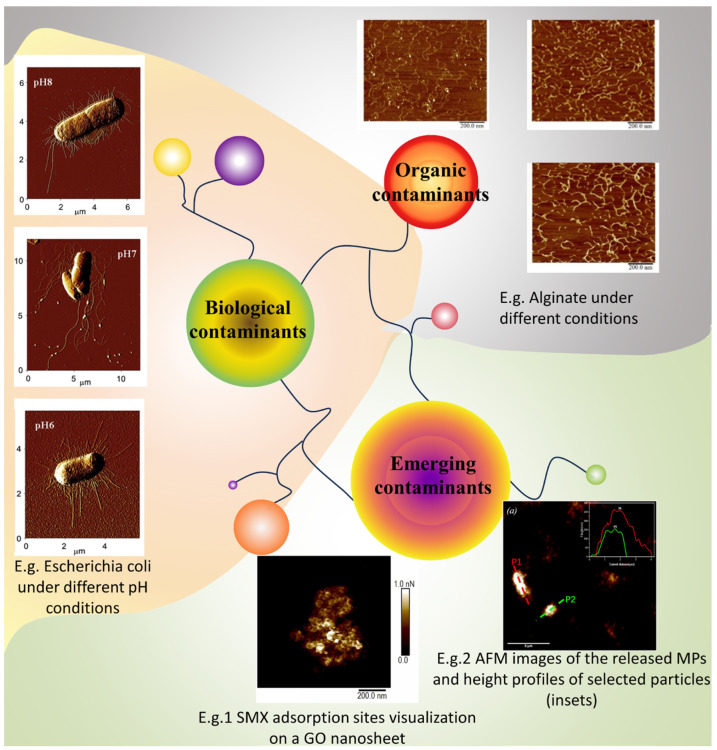
The AFM characterizes different types of membrane foulants, where the AFM images are reproduced from [42,43,44], with permission from Elsevier academic publishing company.

Different types of membrane foulants are encountered in membrane-based technologies and in other techniques; therefore, employing AFM for scrutinizing the characteristics of contaminants at a microscopic level is vital. As shown in Figure 6, the nanometer-scale resolution of AFM enables direct observation of the contaminant morphology and structure on membrane surfaces. Accordingly, AFM could be used to monitor the adsorption and adhesion of contaminants in real-time under various environmental conditions. Morphological changes in living microorganisms during metabolism could be recorded in tapping or non-contact modes, which is a challenge for other techniques. Employing the capabilities of AFM in this way enhances understanding of membrane fouling mechanisms. Characterizing contaminants facilitates superior understanding of membrane fouling principles, and offers essential guidance for the design, operation, and maintenance of membrane filtration systems. This is an important factor in the investigation of membrane fouling mechanisms.

#### 3.2.1. Organic Contaminants

Natural organic matter (NOM) is the primary contaminant in wastewater. It is a complex heterogeneous system comprising diverse organic molecules [45], such as humic substances, polysaccharides, and proteins, which can all affect the membrane performance. Observations using AFM in aquatic environments have revealed that natural polysaccharide sodium alginate (SA) predominantly exists as single helical chains, with diameters of approximately 0.2–0.3 nm [42]. Scanning humic acid sodium (HA)-contaminated mica surfaces with AFM has uncovered spherical particles and aggregates, featuring colloidal diameters under 100 nm and heights from 0.5 to 7 nm [46]. In studies on protein membrane fouling, most protein molecules have been observed as monomers on mica surfaces [47]. Extracellular organic matter (EOM) can lead to severe ultrafiltration membrane fouling. AFM enables the observation of the aggregation and blockage behaviors of pollutants on the membrane surface [48], and evaluates the effects of cleaning/pre-treatment [49]. Utilizing AFM technology aids in further understanding the impact of natural organic matter (NOM) on membrane performance during water treatment processes, thereby laying the foundation for mitigating organic membrane fouling.

#### 3.2.2. Biological Contaminants

In addition to typical organic contaminants, biological contaminants can impair membrane treatment efficiency in water treatment processes. *Escherichia coli* is a common pathogenic microorganism that compromises the safety of water resources and drinking water. Researchers have used AFM to investigate the morphological changes in *E. coli* on membrane surfaces under varying pH conditions [43] and correlate it with membrane filtration and cleaning [50]. In addition, AFM has been used to examine changes in the morphology of antibiotic-resistant *E. coli* on membrane surfaces during photocatalytic Fenton water treatment [51]. Recently, owing to the potential problem of microalgae in water treatment processes, particularly in membrane treatment, AFM has been applied extensively to study microalgal cell morphology and nanomechanical properties on membrane surfaces. High-speed atomic force microscopy (HS-AFM) has been employed to analyze *Chlorella vulgaris* treated with electrocoagulation flotation (ECF) [52]. Another study used AFM to determine the energy required to disrupt individual microalgae cells [53]. Guidance could be offered for alleviating biological fouling caused by microalgae. For the living microbial cells, AFM-based single-cell force spectroscopy (AFM-SCFS) has significant value for characterizing the structure, mechanical properties, and molecular activity of individual living microbial cells [54]. The technique can measure the mechanical properties of a single microorganism, quantify individual microorganism adhesion forces, and perform structural imaging of microbial behavior while simultaneously sensing microbial activity in real-time. Wang et al. [55] employed AFM to explore the dynamic effects of various environmental factors on microorganisms and membrane surface interactions at a molecular scale. This provides a research basis for the effective inhibition of biological foulants on membrane surfaces.

AFM-SFCS allows sensitive measurements of the mechanical properties of individual molecules. This allows researchers to gain insight into the mechanical properties of individual molecules such as stretching, deformation, and fracture, which is important for understanding the properties of biomolecules, polymers, and other materials. Nevertheless, AFM-SCFS has not reached maturity yet and still presents several technical challenges. Based on our group’s research on AFM-SFCS in the environmental field, we found that this technology faces the following problems in its application. First, the adhesion of live single cells to the probe tip is difficult and requires the selection of suitable adhesives for cell immobilization. Additionally, assessing the viability of single cells on the probe tip after attachment is challenging, prompting researchers to explore more advanced methods for examining post-adhesion cell viability. The morphology of single cells is not consistent; it encompasses rod or spherical shapes and other irregular shapes. During the adhesion process, it is crucial to consider different adhesion positions and variations in contact areas with the measurement surface to prevent inconsistencies in the recorded force magnitudes. Furthermore, even when live cells successfully adhere, it is difficult to maintain consistent single-cell activity at the probe tip (considering the different activity levels of young and aged cells at various stages). Ongoing investigation and refinement of AFM-SCFS techniques is anticipated to address these issues in the near future. Researchers could, therefore, gain better understanding of the characteristics of biological contaminants using AFM technology, further elucidate the membrane fouling process, guide biofouling removal, and offer theoretical support and practical guidance for the development of long-lasting antifouling membrane materials and superior biofouling control strategies.

#### 3.2.3. Emerging Contaminants

Emerging contaminants in wastewater treatment processes, such as microplastics, antibiotics, and endocrine-disrupting compounds (EDCs) have been attracting increasing academic attention at national and international levels. As a high-resolution tool, AFM enables a more detailed examination of the physical properties of microplastics [56]. For instance, Melo-Agustín et al. [57] employed AFM for morphological analysis of microplastic surfaces, discovering that polyethylene (PE) microplastic surfaces exhibit higher levels of roughness than polypropylene (PP) microplastic surfaces. This observation suggests that PE is more susceptible to degradation than PP, potentially leading to greater contaminant adsorption. Chen et al. [58] introduced a method that combines AFM with infrared spectroscopy (AFM-IR) to characterize nanoplastics (NPs). This hybrid AFM technique can identify and image the chemical composition of nanoplastics at a high spatial resolution (20–100 nm), thereby offering a novel approach to NP characterization. However, the large specific surface area of microplastics often causes them to function as ‘carriers’ of other contaminants during water treatment processes, which exacerbates pollution. For instance, Zhang et al. [59] employed AFM to determine the interaction forces between NPs (hematite and corundum) and *Escherichia coli* cells, gaining further understanding of the membrane fouling mechanism of microplastics.

Additionally, antibiotics are frequently occurring emerging pollutants in aquatic environments, and even at trace concentrations, antibiotics in wastewater can adversely affect human health. AFM can effectively characterize the morphology and interaction forces of antibiotics on the membrane surface, thereby enhancing the efficiency of membranes in intercepting them. For instance, Liu et al. [60] used AFM to investigate the adsorption of EDCs on nanofiltration membrane surfaces, subsequently enhancing the EDC removal rate by preparing modified nanofiltration membranes. Wu et al. [61] attached sulfamethoxazole (SMX), a representative antibiotic, to an AFM tip to measure the SMX adhesion force distribution. Their study revealed the adhesion mechanism of SMX and, potentially, that of other sulfonamide antibiotics at a molecular level from both experimental and theoretical viewpoints. Researchers have also examined the impact of microplastics on antibiotic transport during sand filtration [44] by grafting ciprofloxacin (CIP) and sulfamethoxazole (SMX) onto AFM probes to determine the adhesion forces between representative microplastics (PS and PE) and quartz sand. Their study explored the mechanism of microplastics that enhances antibiotic transport in sand filtration systems from the perspective of molecular interactions. Table 1 summarizes the entire literature on different aspects of AFM studies of different pollutants in this section. In summary, employing AFM to investigate contaminant morphology under various water treatment conditions contributes to a deeper understanding of the characteristics of the contaminants, which, in turn, could inform the removal of attendant membrane foulants.

### 3.3. Microscopic Identification of Membrane Fouling Processes under Changing Factors

**Figure 7:**
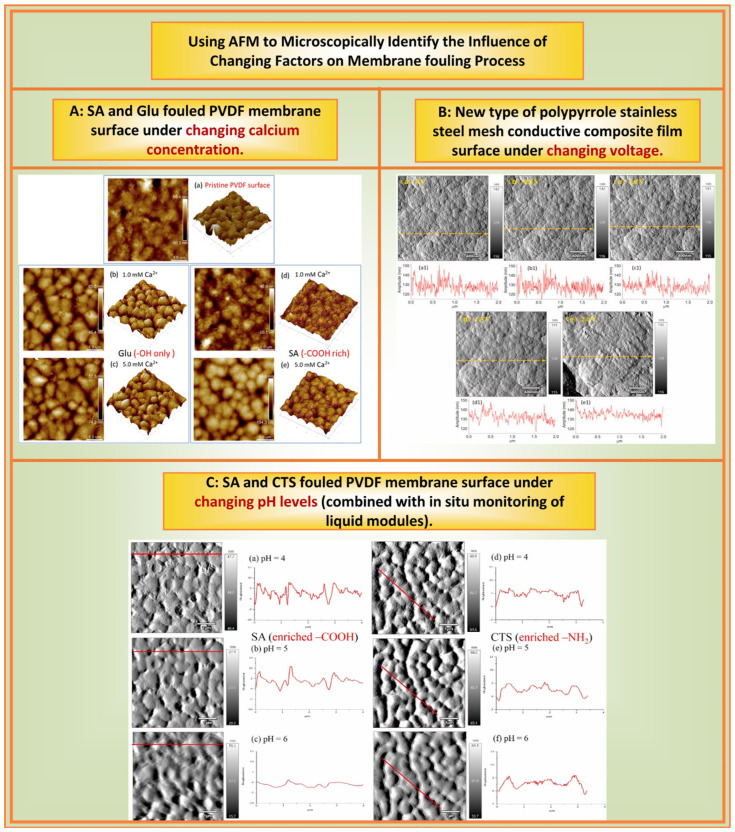
Using AFM to microscopically identify the influence of changing factors on the membrane fouling process, where the AFM images are reproduced from our previous publications [63,64,67].

The previous text, respectively, introduced the membrane and contaminants observed by AFM. However, membrane fouling is a complex fouling process influenced by multiple factors. Therefore, our research team conducted abundant research on the effects of changes in the ionic concentration, pH, and time on membrane fouling using AFM. By employing AFM, researchers can monitor the morphological changes in contaminants under various environmental conditions, facilitating real-time observation of the adsorption process on membrane surfaces. Once contaminants are adsorbed, alterations in environmental factors (ionic conditions, pH, membrane surface properties, and time) could cause varying fouling morphologies and characteristics compared with their initial states. Figure 7 illustrates several aspects of the effect of changing factors on the membrane plugging process using AFM microscopy to identify changes.

Ionic conditions: Our research team [62,63,64,65] used AFM to study the effects of different valence ions on membrane fouling of NOMs. Using AFM force measurements, morphology characterization, and other technical methods, the effect of monovalent ions such as Na^+^ and K^+^ on organic compounds was found to be based on their charge and structure. However, the effect of divalent ions such as Ca^2+^ and Mg^2+^ on organic compounds also included complexation. Among them, it is closely related to the special functional groups, types, and structures of NOMs. Miao et al. [68,69] employed AFM to investigate the effects of Na^+^, Mg^2+^, and Ca^2+^ on HA fouling through HA membrane fouling experiments. These authors observed that membrane fouling intensified at lower Ca^2+^ or Mg^2+^ concentrations and significantly decreased at substantially higher Ca^2+^ or Mg^2+^ concentrations, albeit with the two ions having different mechanisms.

pH: We investigated changes in membrane fouling under different pH conditions using AFM [64]. The results showed that at a pH range of 4–6, the adherence of polysaccharide fouling, and its reversibility, depended on the functional groups. When the organics were rich in –COOH, an increase in pH reduced their deposition on the membrane surface and alleviated adsorptive fouling and irreversibility. For the –NH_2_ functional group, an increase in pH led to more severe polysaccharide fouling owing to a lower degree of protonation, and the resulting fouling was highly irreversible. Modification using GO alleviated the adsorptive fouling of these two polysaccharides on PVDF; however, the extent of alleviation depended on the abundance of functional groups on the polysaccharides.

Time: Interestingly, we found that time changes could affect membrane fouling [55]. We studied the pollution behavior of three selected model foulants at different adsorption times. For the SA-Ca^2+^ system, a longer adsorption time slightly increased the adsorption capacity of SA but significantly reduced its reversibility. With regards to BSA-Ca^2+^, the extended time did not change the amount of BSA deposited on the membrane surface but led to more residual BSA after cleaning. Similarly, in the HA-Ca^2+^ system, the adsorption time had almost no effect on the adsorption amount of HA but reduced its reversibility. Duration had a significant effect on the quantity and reversibility of membrane fouling, depending on the chemical properties of the membrane. Therefore, the AFM measurement results indicate that the longer the adsorption time, the denser the fouling layer and the stronger the interaction force between the fouling membranes.

Other factors: We also used AFM to study the effects of voltage on the fouling of a novel polypyrrole (PPy) and stainless steel mesh conductive composite membrane [67]. We found that the PPy ‘cauliflower’ structure expanded as the applied voltage increased (Figure 7), and the corresponding roughness of the feature area gradually decreased from 5.91 to 4.34 nm. This result could probably be ascribed to the delocalized conjugated electron carrier in the conducting polymer moving along the polymer chain under an external electric field, which changed the dipole moment of the PPy molecules. Such change caused changes in the conformation and intermolecular arrangement of the PPy molecules, resulting in the expansion of surface morphology and, thereby, decreasing the roughness.

In addition to the these membrane fouling investigations, Arkhangelsky et al. [70] employed AFM to investigate the membrane-cleaning process and examined the influence of different cleaning agents on membrane surfaces. Analysis employing AFM revealed that the sodium hypochlorite (NaOCl) cleaning agent affected the contaminants and the membrane, leading to partial organic matter destruction and a modified membrane surface. In contrast, sodium hydroxide (NaOH) treatment completely destroyed the proteins, yielding a smooth surface with minimal residual matter. Similarly, using AFM to examine the fouling behavior of BSA on the membrane, it was found that pre-chlorination significantly mitigated membrane fouling, whereas pre-ozonation oxidation exacerbated it [66]. These studies leveraged AFM technology to characterize the morphology of common contaminants on membrane surfaces and to elucidate the alterations and characteristics of the membrane fouling surface morphology under various conditions, such as time and pH. This information provides a theoretical basis for the mechanism of converting irreversible fouling into reversible fouling, and effectively informs membrane fouling control strategies.

### 3.4. Measurement of Interactions in Membrane Fouling

In the membrane treatment process, the micro-interaction between membranes and foulants significantly affects the formation of membrane fouling. The AFM technology offers valuable insights into the characteristics of foulants and membrane–foulant interactions, which could be leveraged to develop more effective strategies for preventing and controlling membrane fouling. Such strategies include optimizing membrane materials and surface modifications, enhancing pre-treatment processes, and creating innovative cleaning and regeneration technologies, which could reduce operational costs and prolong the lifespan of the membranes. The interaction force between foulants and the membrane is crucial for determining the efficiency of membrane fouling removal. Nanomechanical measurements using AFM and the quantification of interfacial interaction forces during membrane fouling provide essential information on the nanomechanical properties of foulants and membrane surfaces. Such information is critical for understanding membrane fouling.

**Figure 8:**
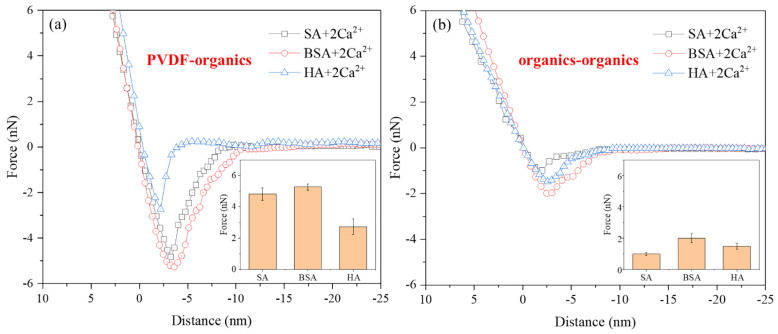
AFM-measured adhesion force change curves for PVDF membrane-organic (**a**) and organic-organic (**b**) interactions under 2 mM Ca^2+^ conditions. Reproduced from our previous publication [74].

The interaction force profiles measured using AFM are shown in Figure 8, where the type of AFM colloidal probe fabricated plays a key role for the interaction force measurements. Combining AFM with BSA-adsorbed SiO_2_ microsphere colloidal probes to investigate membrane surface fouling in the presence of BSA [71]. These authors observed that the adhesion force between PVDF-BSA were −1.5 nN, whereas the adhesion force between BSA-BSA were nearly zero, suggesting that BSA fouling behavior was predominantly influenced by the physicochemical interaction between the membrane polymer and BSA. Membrane-coated colloidal probes made of SiO_2_ microspheres coated with PP/PA are utilized in AFM to investigate the mechanism of membrane fouling caused by HA [72]. Force measurements showed that the interaction between the membrane and foulants was the primary factor contributing to the membrane fouling behavior. In a study of membrane fouling involving HA and SA [73], indention and retraction curves obtained from force spectroscopy measurements using an AFM probe modified with silicon nitride were used to characterize the surface stiffness and adhesive properties of fouled and clean membranes. These authors discovered that bacterial cells neither adhered to nor penetrated the organic fouling layer but, instead, traversed the thin foulant layer and directly adhered to the membrane surface.

To further understand and clarify the fouling behavior of HA and SA on membranes, Miao et al. [75] used AFM in conjunction with PVDF and foulant-coated probes to investigate the intermolecular forces between the membrane and contaminants (SA, HA, or HA/SA mixtures), as well as the forces between the contaminants themselves. Owing to the strong interaction between the hydroxyl groups in SA and PVDF, the adhesion force between PVDF and SA was more than double that of PVDF-HA. The formation of organic fouling on membranes can be studied by adsorbing the corresponding EfOM components onto the surface of PVDF microspheres sintered on cantilevers prepared to form EfOM-coated colloidal probes [76]. Using AFM, these authors demonstrated that the adhesion force between PVDF and different parts of the EfOM follow the order PVDF-TPI (affinitive) < PVDF-HPO (hydrophobic) < PVDF-HPI (hydrophilic). Several researchers have examined membrane fouling under the combined action of BSA and HA [74]. They created colloidal probes with BSA directly attached to the probe tip and employed AFM-based chemical force spectroscopy for adhesion force measurements. Furthermore, employing AFM to examine the interaction energy between polyvinyl chloride (PVC) membranes and three water contaminants, namely HA, BSA, and dextran (DEX) [77], helps in revealing the complex mechanisms of related membrane fouling.

Analyzing the AFM results for the interaction forces between individual and multiple organic contaminants with membranes has led to the following conclusions. Generally, the interaction between membranes and foulants is stronger than the interaction forces among the foulants themselves. HA adsorption significantly decreases the BSA adhesion force on hydrophobic surfaces. The fouling rate of PVC membranes follows the order of DEX > BSA > HA, demonstrating that selecting suitable pretreatment processes to remove specific foulants can effectively control polyvinyl chloride membrane fouling. Owing to the strong interaction between the hydroxyl groups in SA and PVDF, SA, rather than HA, has been identified as the primary cause of PVDF membrane fouling. This implies that the pretreatment process for removing SA is crucial in controlling PVDF membrane fouling. It suggests that employing appropriate methods, such as pretreatment, membrane modification, or cleaning, to reduce the hydrogen bonding interactions between PVDF and foulants is an effective strategy for reducing adhesion forces. Choosing pretreatments that convert HPI and HPO fractions into TPI fractions is essential for controlling PVDF membrane fouling during secondary effluent filtration. Therefore, AFM force measurements provide valuable information for selecting membrane modifications, feedwater pretreatment, and cleaning technologies in wastewater treatment and desalination.

Recently, AFM has increasingly been employed to investigate the interaction forces between various foulants and membranes. Single force spectroscopy curves from AFM are used to assess the interactions between membranes and foulants, serving as a crucial parameter for adjusting the properties of modified membranes. This technique can measure not only the interaction forces between hard objects but also those involving softer entities, such as in the interaction force measurements between pretreated modified membranes and related membrane foulants [78]. Scholars have used AFM’s single force spectroscopy curves to elucidate the mechanism of scaling in electrodialysis induced by anionic polyacrylamide (APAM) in anion exchange membranes (AEM) [79]. AFM can also characterize the interaction forces between various coatings and other substances, apart from membranes, such as the interactions between bubbles in different solutions [80], dissolved organics [81], and spherical particles of asphalt coating. It can also measure the interactions between living microorganisms and membranes, which is vital since living cells, being alive, secrete exosomes under external forces and their interaction forces with membranes change under stress conditions. Using AFM to measure these forces can more accurately reflect the biological fouling on membrane surfaces. Yumiyama and others [82] directly measured the interaction forces between individual yeast cells. Scholars have also studied the adhesion forces between yeast cells and microbubbles (MB) [83]. These studies demonstrate the utility of AFM’s single force spectroscopy curves in measuring the interaction forces between foulants and membranes, which can be used to evaluate the characteristics and interactions at the membrane–foulant interface. This aids in developing high-performance modified membranes and more effective membrane cleaning methods.

### 3.5. Modeling or Analysis of the Interaction in Membrane Fouling

In understanding membrane fouling processes, the characteristics of impurities (such as size, shape, charge properties, and chemical stability) and the attributes of membrane materials (such as pore size, surface roughness, chemical stability, and charge properties) significantly influence the interaction modes between impurities and the membrane. Certain impurities could interact more strongly with specific membrane materials, potentially leading to severe membrane fouling. For example, positively charged impurities could be strongly adsorbed onto negatively charged membrane materials, forming a fouling layer. Conversely, if repulsive forces are generated between the membrane material and the impurities because of their charges, the degree of fouling could decrease. Ionic composition can also have a significant impact on foulant–foulant interactions [84]. Therefore, the modeling and analysis of such interactions could provide key insights for predicting and optimizing the performance of membrane processes.

By combining the results of AFM force measurements with certain existing theories or models, such as the extended Derjaguin–Landau–Verwey–Overbeek (XDLVO) theory [85,86] and the Hermia model [87,88], it is possible to predict the manner in which forces act as particles approach the membrane surface, as well as their impact on particle adsorption behavior. This allows for the prediction of membrane fouling based on molecular characteristics. Wang et al. [62] employed the XDLVO model to calculate the interaction energy between PVDF membranes and organic matter under different ionic strengths, finding that as the Na^+^ concentration increased, the Lewis acid–base (AB) force values gradually decreased. The AB forces are related to the chemical functional groups of the particles and the membrane [89]. This result shows that an increase in ionic strength enhances the AB interaction between the membrane and organic matter, which is consistent with the total amount of organic matter adsorbed on the membrane surface as the ionic strength increases. Not only that, XDLVO interactions and surface roughness may collectively influence the transport and fate of emerging multifunctional nanohybrids in the environment [90]. In addition, these authors [91,92] found that the results calculated by the XDLVO theory aligned with the AFM analysis results, suggesting that AFM force–distance curves could effectively validate the calculated results and that AFM is highly reliable for measuring the interactions between the membrane and foulants. Integrating AFM force measurement techniques to analyze blocking mechanisms during membrane filtration [87] aids in better understanding the phenomenon of membrane fouling and in developing effective fouling prevention strategies.

The Hermia model [88], by fitting the relationship between apparent fouling resistance and membrane filtration time, identifies the types of fouling caused by different blocking mechanisms [93]. Huang et al. [94] developed the Unified Membrane Fouling Index (UMFI) based on the Hermia model. By directly testing commercial membranes, UMFI can quantify the likelihood of membrane fouling, which is very useful for evaluating fouling observed in low-pressure membranes (LPMs) across different water treatment scales. AFM force measurement technology can be used to validate these established models, helping to deepen the understanding of membrane blocking mechanisms.

It should be noted that although the XDLVO theory and Hermia model provide useful insights, they do not encompass all types of fouling behavior. These theories and models are more suitable for predictions under steady conditions, whereas actual water treatment processes are conventionally confronted by more complex, dynamic, and changing conditions. New research seeks to integrate experimental and theoretical approaches for a more comprehensive understanding and prediction of the interactions between impurities and membrane materials. For example, AFM and other nanoscale characterization techniques are used for direct observation and measurement of the interactions between impurities and membranes, whereas molecular dynamics simulations and quantum chemical calculations are used to understand these processes at the atomic scale. Analyzing and modeling the potential interactions between different impurities and membrane materials is a key factor in membrane science and engineering. Integration of various experimental and theoretical methods is required to gain a comprehensive and in-depth understanding. In this process, AFM could predict the adsorption tendency of pollutants by measuring the interaction forces between the pollutants and the membrane surface. Further, AFM could also monitor the fouling process, such as adsorption, diffusion, and aggregation of pollutants on the membrane surface in real-time. Combining AFM with relevant theories and models helps to further the exploration of the membrane fouling process and the prediction of membrane fouling trends.

## 4. Application of Improved AFM Technology Membrane Fouling Research

As technology continues to progress, improved AFM techniques have attained greater precision, faster scanning speeds, wider application scopes, and additional functionalities. Improved AFM technology has significant potential in membrane fouling research by providing high-precision characterization data and enabling in situ monitoring and dynamic analysis. This ability offers more scientific and accurate support for the prevention and control of membrane fouling.

### 4.1. Modification of Probes for Membrane Fouling Characterization

**Figure 9:**
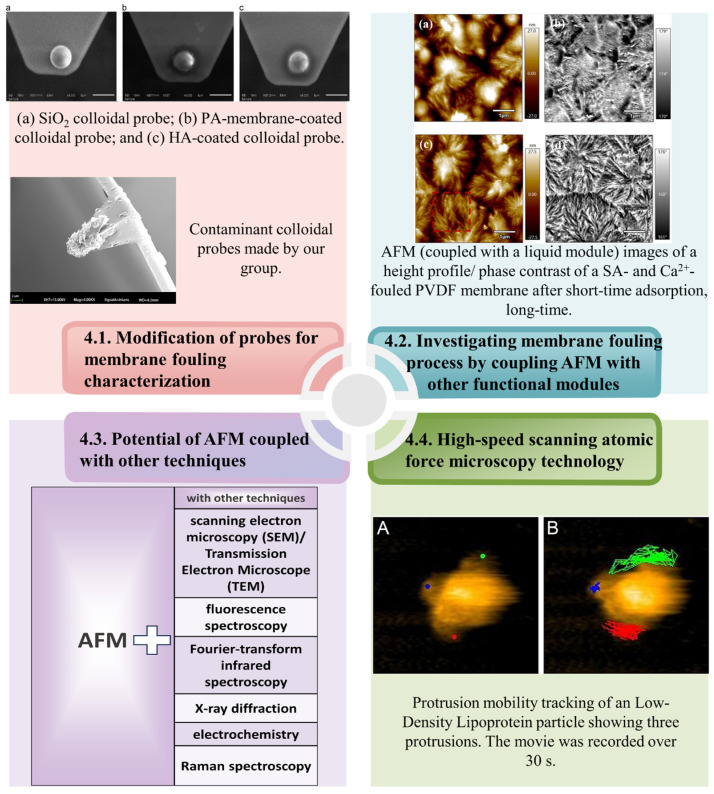
Application of improved AFM technology membrane fouling research, where the AFM images are reproduced from [55,72,95], with permission from Elsevier academic publishing company.

From the initial 10-nanometer resolution to the current sub-angstrom level, researchers have enhanced the resolution of microscopes. This was achieved by optimizing the scanning probes and increasing the AFM scanning speeds by enlarging the scanning head sizes and using higher resonance frequencies. Continuous advancements have also rendered AFM more sensitive for mechanical detection, facilitating the determination of the local mechanical properties of materials at a nanoscale. These enhancements rely mainly on cutting-edge AFM probes. The AFM probe tip is a critical component, and its performance directly influences the precision and reliability of AFM measurements. Three primary methods are used for preparing colloidal probes to investigate membrane fouling. These are attaching modified contaminant particles to the probe tip for force measurement, directly modifying the contaminants, and using an adhesive to adsorb the contaminants for measurement. As shown in Figure 9 (4.1), our research team utilized bioadhesives such as dopamine to directly attach contaminants to the AFM probe tip, resulting in a colloidal probe for AFM force measurements. The foulant colloidal probes were prepared using AFM with the Cypher ES. The organic foulants microspheres came in powder form, were ground using a ball mill (leading to a particle size of approximately 0.2 μm), and then filled uniformly into microplates (pore size of 5 μm). The photosensitive glue (A332) was filled evenly on the other side of the microplate. Then, the microplate was fixed on the AFM platform. The cantilever was lowered to adhere the glue, then lifted it to the other side to adhere the organic foulants. The adhered needle tip was irradiated with a UV lamp for 30 s to achieve quantitative modification with organic foulants. This method decreases the contact area to approximately 5 μm, leading to more accurate and reliable measurement results.

Many researchers have made efforts in modifying colloidal probes. Fleischmann [96] first employed AFM to quantitatively define the 3D shape of atomic probe tips, opening new possibilities for studying the physical mechanisms in (laser-assisted) atomic probes. Owing to the complexity of the sample surface morphology and composition, modified AFM probe tips with varying surface chemical affinities could enhance selectivity, ensuring more accurate and precise measurements in specific applications; as shown in Figure 9 (4.1), utilizing AFM in conjunction with custom-modified membrane-coated and HA-coated probes to assess the adhesion forces between membrane-HA and HA-HA [72]. Initially, this demonstrated the potential application of modified AFM colloidal probe microinterface force measurements for UF membrane fouling behavior. Additionally, the AFM tip can be modified by incorporating different representative organic functional groups [97], namely benzyl, hexyl, propionic acid, ethylamine hydrochloride, and propionic acid propyl ester. These authors measured the adhesion forces between the modified AFM tips and reverse osmosis membranes to determine the potential scaling tendency of each functional group category on the membrane. To enhance the accuracy of AFM force measurement data, Nguyen et al. [98] employed four distinct AFM probes to gauge the nanomechanical properties of three different samples, providing valuable insights for probe selection for better interpretation of force indentation data. Furthermore, the use of modified tips broadens the applicability of AFM measurements. By attaching a mineral particle to a tipless AFM cantilever, a mineral probe for AFM measurements can be created and, afterward, applied an atomic force microscope equipped with pyrite or chalcopyrite tips to investigate the adhesion of thermophilic thiosulfate-oxidizing bacteria [99]. The modified AFM tips significantly enhanced the accuracy and reliability of the AFM measurements, reduced the probe replacement frequency, and rendered them suitable for a more extensive range of applications. Modified colloidal probes could achieve in situ measurements of the interaction forces between membranes and foulants under varying ion concentration conditions. This ability provides a valuable research method for measuring membrane–foulant interactions in wastewater treatment.

### 4.2. Investigating Membrane Fouling Process by Coupling AFM with Other Functional Modules

When studying membrane fouling processes, the liquid module of AFM could be used to change the solution environment and conduct in situ AFM measurements of the membrane morphology and membrane pollutant interaction forces. Moreover, AFM encompasses various functional modules that could be employed to investigate membrane fouling phenomena under diverse conditions.

Generally, researchers use an open module to examine the membrane fouling process in an air environment. However, given the complexity of membrane−fouling environments, AFM could be integrated with multiple functional modules to conduct research under various environmental conditions. The chemical environment of a solution is crucial in membrane fouling. Coupling AFM with a liquid cell module enables researchers to perform AFM measurements in water and other solvents, which is important for studying membrane fouling in actual treatment processes. Using a micropump, chemical solutions with altered conditions (e.g., pH, concentration, and ion concentration) can be introduced into the system, allowing real-time AFM monitoring of the membrane surface morphology and roughness changes as the solution conditions vary [64]. As shown in Figure 9 (4.2), This approach facilitates simulation of actual water treatment environments, enabling dynamic observation of membrane conditions as the properties of the chemical solution change, and provides more precise in situ observations of contaminant adsorption and attachment processes on the membrane surface.

Additionally, the add-on FAST module can independently acquire probe signals [100] and can be installed without modifying the existing scanner hardware or electronic equipment. This module facilitates seamless switching between fast and slow scanning modes, contributing to a clearer and more comprehensive understanding of membrane fouling. By incorporating an electrochemical module [101], researchers can observe electrochemical processes on the membrane surface, which is invaluable for examining chemical reactions and ion migration processes during membrane fouling. Temperature significantly affects the contaminant adsorption and attachment processes on the membrane surface, with distinct fouling characteristics and interaction mechanisms between contaminants and the membrane surface at different temperatures. Integrating high- and low-temperature modules with AFM enables measurements under various temperature conditions. These AFM modules offer numerous experimental methods and conditions for research on membrane fouling, contributing to a deeper understanding of the occurrence, development, and impact of membrane fouling. By applying these modules, researchers can provide robust support for the improvement and development of membrane filtration technologies.

### 4.3. Potential of AFM Coupled with Other Techniques

Combining AFM with other imaging and spectroscopic techniques could provide comprehensive information regarding membrane fouling. Typically, as shown in Table 2, AFM provides high-resolution surface morphology information and, when integrated with scanning electron microscopy (SEM) or transmission electron microscopy (TEM), SEM and TEM offer structural and elemental composition information, resulting in more comprehensive characterization [102,103]. Combined with fluorescence spectroscopy, chemical composition information is provided [104], which is useful for studying the properties of biological and other organic membranes. Integration with Fourier-transform infrared spectroscopy (FTIR) [105] produces information on chemical composition, which enables in situ analysis of the molecular structure, bonding, and distribution on the membrane surface, and further reveals the chemical characteristics and mechanisms of membrane fouling. Coupling with X-ray diffraction (XRD) [106] provides information on the crystalline properties of inorganic membranes.

Currently, although electrochemical atomic force microscopy (EC-AFM) is widely used in the field of materials [107], its potential in the field of studying membrane contamination should not be overlooked. EC-AFM can initiate electrochemical reactions by applying an external potential to the scanning probe, allowing AFM to observe electrochemically active regions on the surface and collect scanning images to study the local chemical reaction behavior, polarization phenomena, and impurity deposition processes on the membrane surface. This ability offers an intuitive understanding of the morphological changes and evolution of impurities on the membrane surface [101], as well as a highly effective means of exploring membrane fouling mechanisms. Moreover, the technique offers important guidance and a basis for designing novel anti-fouling membranes and technologies for membrane cleaning. Both AFM and Raman spectroscopy are effective for characterizing material surface properties [109]. While AFM provides information on surface morphology, roughness, and nanomechanical properties, Raman spectroscopy provides chemical composition and structural information. Using AFM facilitates the observation and analyses of the adsorption and adhesion processes of surface contaminants, as well as understanding of the morphological features of the contaminants on the membrane surface. Raman spectroscopy enables obtaining chemical information about the contaminants on the membrane surface, identifying the types and structures of contaminants [108], furthers understanding of the interaction mechanism between contaminants and membrane surfaces, and provides guidance for the optimization of membrane filtration systems. A combination of AFM and Raman techniques [110] provides more comprehensive and accurate information for membrane fouling research, helping researchers delve deeper into membrane fouling mechanisms, and offers robust support for the improvement and development of membrane filtration technologies.

In summary, AFM can be combined with other imaging and spectroscopic techniques to provide more comprehensive data and deeper understanding. These improvements in AFM technology and analytical methods have further refined AFM technology, presenting new possibilities and ideas for its application. In the future, AFM technology will be applied more widely in the membrane fouling and water treatment fields, providing development support and assurance, and facilitating further scientific research.

### 4.4. High-Speed Scanning Atomic Force Microscopy Technology

The high-speed version of AFM (HS-AFM) is an innovative imaging technique that surpasses traditional AFM in speed [111]. This technique employs a non-resonant probe, and the distance between the probe and sample can be adjusted in real-time, enabling ultrafast scanning and imaging, with scanning rates exceeding a thousand pixels per second.

Research on HS-AFM related to membrane fouling is advancing progressively. Because of its high-speed scanning and high spatial resolution capabilities, HS-AFM can swiftly and accurately observe and image membrane surfaces, providing new tools and platforms for exploring membrane fouling mechanisms and studying anti-fouling technologies. Further, HS-AFM can be used to track the adhesion behavior and evolution of contaminants on a membrane surface in real-time. As shown in Figure 9 (4.3), by employing high-speed scanning technology, HS-AFM can record dynamic changes in membrane surface contaminants with high temporal resolution (millisecond level), including the morphology, size, and density of the contaminants. Thereby, improved understanding is facilitated of the physical behavior and fouling mechanisms of contaminants. In addition, HS-AFM could be used to study the adhesion, diffusion, and reaction processes of contaminants at a molecular level [112], such as measuring the changes in the interaction forces between membrane surface contaminants and anti-fouling membranes. This information is important for designing more efficient anti-fouling membranes and membrane-cleaning technologies. In conclusion, as an emerging high-speed imaging technique, HS-AFM is being developed and improved continuously [113], and is anticipated to uncover new avenues for investigating membrane fouling mechanisms and anti-fouling technologies in the future, which, ultimately are crucial for advancing membrane cleaning.

## 5. Conclusions and Perspectives

Related studies are increasingly being conducted and people are increasingly using AFM to explore membrane fouling. Utilizing AFM for microscale investigations of the interfaces and interactions in such fouling processes helps reveal the mechanisms of fouling and optimize control strategies. However, relatively few studies have comprehensively reviewed the application of AFM in membrane fouling research. This review delineates the in situ capabilities of AFM in fouling characterization, covering aspects such as the membrane and foulant properties, effects on the cleaning of fouling, and the fouling interaction forces, thereby facilitating quantitative understanding of the nature of fouling. The findings of the current study should help readers gain a deeper understanding of classical AFM applications.

Here, we discussed the application of AFM to characterize and quantify membrane fouling, which highlights the capabilities of AFM for delineating surface roughness, membrane morphology, and contaminants. Observations from AFM suggest that higher membrane surface roughness typically correlates with more severe fouling. By utilizing AFM, fundamental phenomena on the membrane surface could be observed, allowing accurate predictions of fouling trends. Additionally, detailed inspections of contaminant morphologies using AFM further elucidated the membrane fouling process, especially when employing AFM-based AFM-SCFS to characterize the structure, mechanical properties, and molecular activities of individual living microbial cells. Reflecting on the current state of AFM-SCFS research, this study identifies the challenges faced by the technology and offers both theoretical foundations and practical guidelines for developing long-lasting antifouling membrane materials and strategies for membrane fouling control. This study presents examples of using AFM to discern the impact of factors such as salt on the membrane fouling process. Our research team conducted a series of experiments by varying parameters such as salt solution concentration, pH, duration, and ionic states. These experiments could help readers reach a deeper understanding of classical AFM applications and enable interpretation of the associated scientific phenomena that could be encountered.

This article consolidates numerous AFM employment cases to measure the interaction forces associated with membrane fouling. Based on our findings, we proposed guidelines for membrane modification and cleaning processes. The characteristics of impurities and membrane materials profoundly influence their interaction patterns. Specific impurities could similarly interact with specific membranes. This study reviewed existing theories and models of surface interactions in membrane fouling. However, few studies have proposed novel models for membrane fouling. In the future, artificial intelligence (AI) or deep learning could be employed to establish datasets from AFM interaction force measurements. Such datasets, integrated with current theories and models, could pave the way for innovative modeling techniques, providing a more holistic understanding of the interactions between impurities and membrane materials. With ongoing advancements in pivotal technologies, sophisticated AFM techniques are expected to play a broader role in membrane fouling research. This study outlines the current state of modified probes used in membrane fouling measurements. Based on the findings of our research group, we elucidated the probe modification process. Owing to its simplicity and ease of operation, along with the advantage of reduced contact area of the modified probe tip, this method is particularly beneficial. The integration of AFM with other functional modules and technologies is also discussed, offering greater prospects for exploring membrane fouling, particularly in situ investigations of fouling under the influence of salts or other factors by using a liquid module combined with AFM, which proposes a viable detection method for future studies.

In the future, advancements in AFM technology will involve the optimization of probe materials (selecting probes with varying properties based on the materials being tested, differing hardness of materials used in probe fabrication results in distinct piezoelectric effects, leading to diverse measurements of membrane surfaces, contaminant surfaces, and membrane fouling morphology and roughness) and the development of new analytical methods (pore construction technology and a novel approach combining the roughness index with root mean square roughness for more accurate analysis of roughness). These improvements will enhance the ability of AFM to control sample surface morphology and roughness. Further exploration of AFM-modified tips will lead to the incorporation of additional techniques in the construction and modification of colloidal probes, facilitating the acquisition of membrane information under various pollution conditions and providing additional methods for measuring the interfacial interactions between contaminants and between membranes and contaminants. The development of high-speed AFM will enable significant increases in scanning speed during sample scanning, allowing for the dynamic observation of membrane fouling processes. As different functional modules are developed, future AFM systems will couple with various modules (e.g., liquid cell, electrochemical, high and low temperature, and FAST), enabling clearer investigations of membrane fouling under specific environmental conditions. Additionally, innovative combinations of AFM with other techniques could be pursued, such as integrating AFM with infrared spectroscopy, using a reflective crystal to create a modified tip with an attenuated total reflection (ATR) infrared attachment for simultaneous measurement of membrane surface morphology and functional groups, or combining AFM with tip-enhanced Raman spectroscopy (TERS) to improve the microstructure characterization potential. These in-depth explorations and innovations will increase the multidimensional resolution and comprehensiveness of research, enabling the acquisition of more detailed and multidimensional data, enhancing the depth and thoroughness of investigations, and expanding the application scope of AFM technology to interface characteristics.

## Figures and Tables

**Table 1:** Summary of research on AFM applications in contaminants.

Research Content	AFM Model	Characterization Properties	Results	Usage Patterns	Reference
Sodium alginate (SA)	Bruker AXS Multi-mode 8, Madison, WI, USA	Morphology	Alginates exist in single coiled chains	Contact mode	[42]
Effect of Na^+^ on organic fouling	Cypher ES, Oxford Instruments Asylum Research, Abingdon, UK	Morphology and interaction force	/	/	[62]
Effect of carboxyl and hydroxyl groups on adsorptive polysaccharide fouling	Cypher ES, Oxford Instruments Asylum Research, Abingdon, UK	Morphology	Transformation from ‘egg box’ model to formation of network gel	/	[63]
Effects of –COOH and –NH_2_ on adsorptive polysaccharide fouling	Cypher ES, Oxford Instruments Asylum Research, Abingdon, UK	Morphology and interaction force	In pH range 4–6, adherence of polysaccharide fouling and its reversibility depend on the functional groups	Tapping mode	[64]
Effect of sodium and potassium on polysaccharide fouling on PVDF and graphene-oxide-modified PVDF membrane surfaces	Cypher ES, Oxford Instruments Asylum Research, Abingdon, UK	Interaction force	SA fouling in Na^+^ condition more severe than that in K^+^ owing to higher attraction forces under identical ion strengths	Tapping mode	[65]
Humic acid (HA)	Nanoscope IIIa SPM, Digital Instruments, Goleta, CA, USA	Morphology	Spherical particles and aggregates are found with apparent colloidal diameters < 100 nm and heights ranging from ~0.5 to ~7 nm	Tapping mode	[46]
Effect of Na^+^ and Mg^2+^ on adsorptive humic acid fouling	MultiMode 8.0 AFM (Bruker, Ettlingen, Germany)	Interaction force	Cations mainly affect HA fouling by controlling electrostatic and hydration forces of membrane–HA and HA–HA	Contact mode	[66]
Effect of Ca^2+^ and Mg^2+^ on adsorptive humic acid fouling	MultiMode 8.0 AFM (Bruker, Ettlingen, Germany)	Interaction force	Mitigation mechanisms differed for both ions	/	[67]
Bovine serum albumin (BSA)	/	Morphology	Most protein molecules are spread onto mica surface as monomers	Tapping mode	[47]
Effect of chlorination and ozonation on adsorptive protein fouling	MultiMode 8.0 atomic force microscope (AFM, Bruker, Ettlingen, Germany)	Interaction force	BSA fouling definitively mitigated by pre-chlorination but enhanced by pre-ozonation	Contact mode	[66]
Flagellar morphology of *E. coli* cultured at different pH conditions	Nanowizard AFM (JPK Instrument, Berlin, Germany)	Morphology	Differences in flagellar morphology at different pH values	Contact mode	[43]
*E. coli* under action of different disinfectants	Digital Instruments Veeco Metrology Group, Santa Barbara, CA, USA	Morphology	Differences in cell morphology under action of different disinfectants	Tapping mode	[50]
Changes in cell morphology of antibiotic-resistant *E. coli*	Asylum Research Cypher AFM (Oxford Instruments, Abingdon, UK)	Morphology	Damage to *E. coli* cells eventually leads to cell lysis	/	[51]
Different types of MPs	AFM diMultiMode V (Veeco, San Jose, CA, USA)	Morphology and roughness	Different types of MPs have different characteristics	/	[57]
Combined AFM and infrared spectroscopy IR (AFM-IR) characterization of MPs	/	Morphology and roughness	/	/	[58]
Forces between two NPs and *E. coli*	Agilent 5500 AFM (Molecular Imaging, Phoenix, AZ, USA)	Interaction force	Particle sizes of both hematite (α-FeO) and corundum (α-AlO) NPs significantly affected the strength of the adhesion force	Contact mode	[59]
Changes in hydrogel occurring when algae are present in the culture	HS-AFM, Bristol Nano Dynamics Ltd., Bristol, UK	Roughness	Roughness on the algal flocs significantly more pronounced than in the hydrogel layer	Contact mode	[52]
*Clostridium perfringens* treated by electrocoagulation floatation (ECF) method	AFM (Ntegra with Solaris platform, manufactured by NT MDT, Moscow, Russia)	Interaction force	Inefficiency of mechanical cell crushing process	Tapping mode	[53]

**Table 2:** Potential of AFM coupled with other techniques.

Atomic force microscope (AFM)	**with** **Other Techniques**	**Results**	**References**
	Scanning electron microscopy (SEM)/ Transmission electron microscope (TEM)	Provides high-resolution surface morphology information with structural and elemental composition information	[102,103]
	Fluorescence spectroscopy	Provides high-resolution surface morphology information with chemical composition information	[104]
	Fourier-transform infrared spectroscopy	Provides high-resolution surface morphology information with chemical composition, which enables in situ analysis of the molecular structure, bonding, and distribution on the membrane surface	[105]
	X-ray diffraction	Provides information on the crystalline properties of inorganic membranes	[106]
	Electrochemistry	Allows AFM to observe electrochemically active regions on the surface and collect scanning images to study the local chemical reaction behavior, polarization phenomena, and impurity deposition processes on the membrane surface	[107]
	Raman spectroscopy	Provides chemical composition and structural information	[108]

## Data Availability

No new data were created or analyzed in this study. Data sharing is not applicable to this article.
